# Brachial Artery Injury in a Child following Closed Elbow Dislocation: Case Report of a Rare Injury

**DOI:** 10.5704/MOJ.1611.001

**Published:** 2016-11

**Authors:** K Vickash, A Amer, A Naeem, S Falak

**Affiliations:** Department of Orthopaedic and Spine Surgery, Ghurki Trust Teaching Hospital Lahore, Pakistan; * Department Of Plastic and Hand Surgery, Ghurki Trust Teaching Hospital Lahore, Pakistan

**Keywords:** Elbow dislocation, brachial, vascular injury, child

## Abstract

Elbow dislocation, though a common orthopaedic emergency is rare with brachial artery injury and is even more uncommon in the paediatric age group. We present the case of a child who sustained trauma resulting in closed elbow dislocation with brachial artery injury. Elbow dislocation with brachial artery injury can present with palpable distal pulses and good capillary refill because of rich collaterals at the elbow. But this patient presented with signs of frank ischemia distally, and was managed with ipsilateral reverse cephalic vein graft. He had good volume pulses at one year follow-up. Patients with such presentation should have careful clinical and radiological assessment to exclude complicated elbow dislocation.

## Introduction

Elbow is second common site for dislocation after shoulder. Though its location is close to neurovascular bundle but vascular injury is quite rare around elbow. Very few cases have been reported since then with different management and outcomes. Brachial artery injury associated with closed elbow dislocation is rare, especially in the paediatric age, with reported incidence of only 0.3 to 1.7%^1^. Brachial artery injury following blunt elbow trauma is well reported injury after supracondylar fracture in children^2^. Despite extensive collateral circulation, many investigators have also expressed concern about the possible consequences of decreased distal perfusion after ligation of the brachial artery, including cold intolerance, claudication of the forearm and hand, and gangrene^3^.

## Case Report

We present the case of a 10 years old male child with a history of fall from a tree and landing on his right outstretched hand. He presented at our centre one hour after the injury with pain and swelling of the right elbow. On examination the overlying skin was intact, with bruises in the antecubetal fossa, swelling, deformity and tenderness of the elbow. Brachial, radial and ulnar arteries were not palpable. The extremity was cold with a poor capillary refill. There was no sensory or motor loss.

**Fig. 1:**
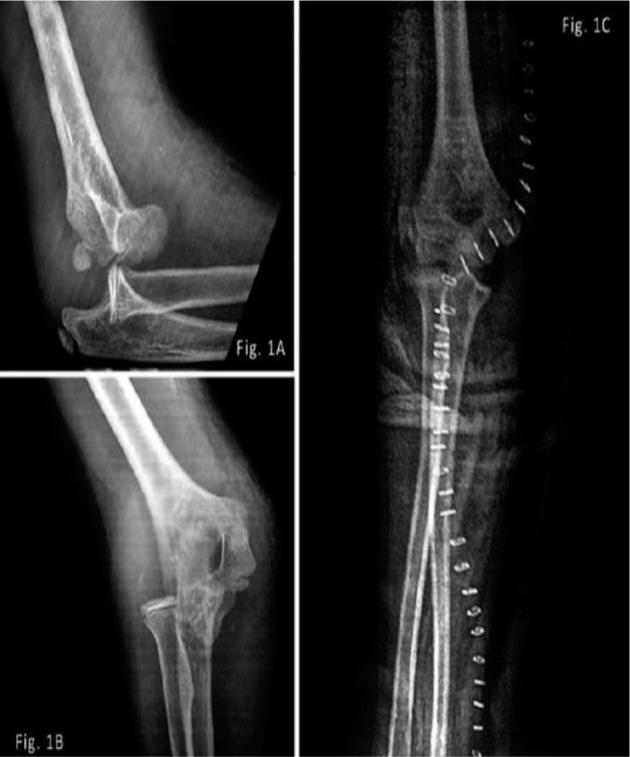
1A, 1B showing posteromedial dislocation of the elbow Fig. 1C post-reduction radiograph showing curvilinear incision closed with staples.

Radiography showed posteromedial dislocation of the elbow (Fig. 1A, 1B). No blood flow was detected beyond the elbow with Doppler ultrasound (U/S).

**Fig. 2:**
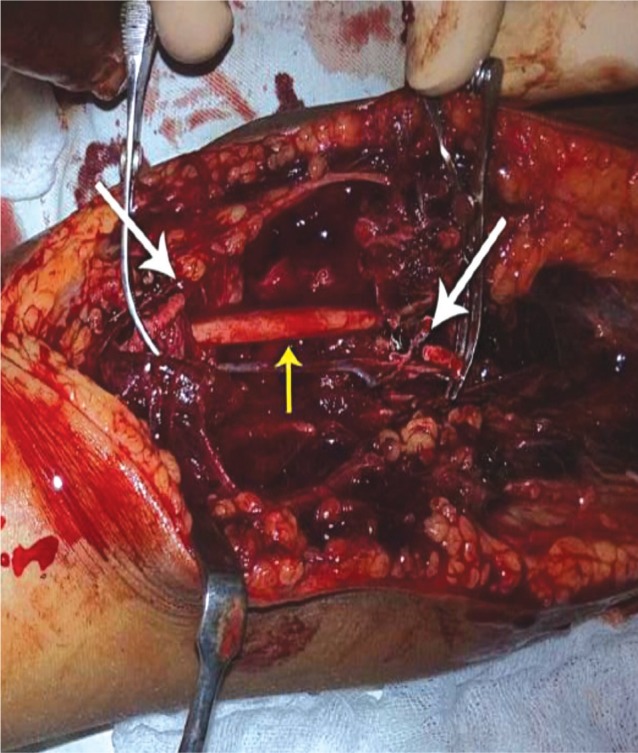
Exploration of the elbow, white arrows indicating transected ends of the brachial artery and yellow arrow indicating Median Nerve.

**Fig. 3:**
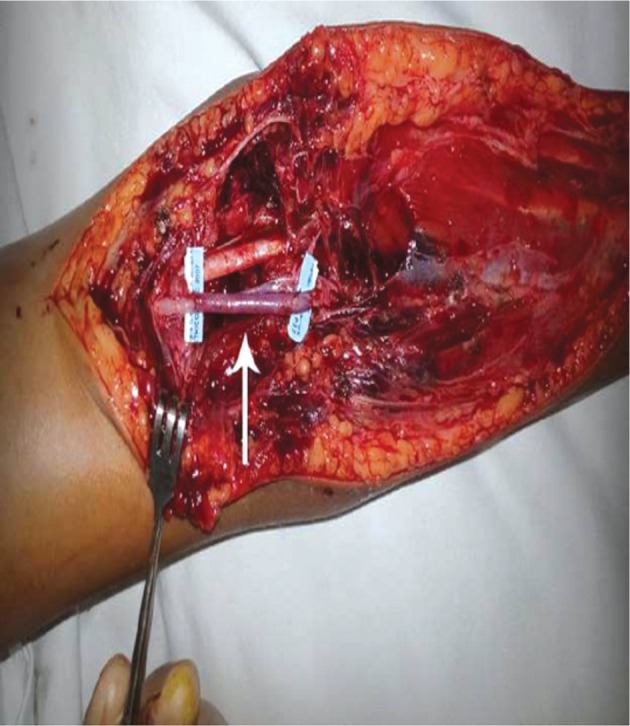
Showing the transected brachial artery with reverse cephalic vein graft indicated by the blue tags.

Elbow was reduced with closed reduction maneuver i.e. elbow flexed, arm suppinated and inline traction. Patient had repeated examination of the peripheral pulse to assess vascular status but no pulse was palpable. Parents were briefed about the situation and informed consent obtained. In the operation theatre the elbow was opened via an anterior approach with curvilinear incision. There was a hematoma and soft tissue oedema and the brachial artery was found transected at the proximal border of pronater teres, with the ends of the artery lying 5cm apart (Fig. 2). The artery was repaired using ipsilateral reverse cephalic vein graft using 6/0 polypropylene sutures (Fig. 3). The elbow was found to be stable after reduction with no evidence of ligamentous injury. A posterior splint was applied with the elbow flexed at 90°. (Fig. 1C)

Post reduction radiographs were found acceptable. The splint was removed at two weeks. At one year follow up the patient was having good volume pulses in the radial and ulnar arteries, full range of elbow motion without any instability.

## Discussion

A large case series study conducted recently reported only one case of brachial artery injury with elbow dislocation in a child, with most of the vascular injuries being in supracondylar fractures. Brachial artery is vulnerable to injury at its distal end inposterior dislocation of the elbow, in which the brachial artery is entrapped between the rigid bicipital aponeurosis and the dislocated distal humerus.

Diagnosis of brachial artery injury is usually missed or delayed as it is an uncommon complication of elbow dislocation. Besides, the upper limb has a rich collateral flow through the profunda brachii, radial and ulnar collateral arteries which may mask the signs of the injury^3^. In such situations the patient may present with subtle clinical features of low volume pulse, poor capillary refill and low oxygen saturation on pulse oximeter. Guler mentioned a case with the brachial artery disruption in closed elbow dislocation in which diagnosis was delayed as the hand was well perfused and radial pulsation was present initially. So, one should have repeated vascular examinations to exclude any vascular injury at the initial presentation^4^.

Because of the rich collateral circulation at the elbow joint frank ischemia following brachial artery injury is rare. In our case, patient had clear clinical features of frank ischemia and so emergency surgical intervention was undertaken. Angiography is recommended in such situations to determine the site and extent of injury and to plan surgical management. But sometimes this facility is not readily available in emergency situation. Doppler ultrasound does provide a reasonable alternative within a short time and at low cost^5^.

Vascular injury can range from complete, sub-adventitial rupture, incarceration and thrombosis. Direct suturing is feasible in cases of clean sections: alternatively reverse shunting with great saphenous or reverse cephalic graft repair^1^.

Fasciotomy is indicated in case of severe soft tissue injury, raised intra-compartmental pressures of forearm and extended delay in the repair (of more than four hours)^3^. Fasciotomy was not performed in our case due to timely definitive management of the problem.

Brachial artery injury with elbow dislocation is rare, especially in the paediatric age group. One should conduct careful clinical and radiological assessment of the patient to exclude complicated elbow dislocation. Any vascular injury with ischemia should be managed with urgent surgical intervention and vigilant follow up.
